# Tunable Electronic and Topological Properties of Germanene by Functional Group Modification

**DOI:** 10.3390/nano8030145

**Published:** 2018-03-06

**Authors:** Ceng-Ceng Ren, Shu-Feng Zhang, Wei-Xiao Ji, Chang-Wen Zhang, Ping Li, Pei-Ji Wang

**Affiliations:** School of Physics, University of Jinan, Jinan 250022, China; renceng2011@163.com (C.-C.R.); sps_zhangsf@ujn.edu.cn (S.-F.Z.); sps_jiwx@ujn.edu.cn (W.-X.J.); ss_zhangcw@ujn.edu.cn (C.-W.Z.); ss_lip@ujn.edu.cn (P.L.)

**Keywords:** germanene, functional group, external strain, topological insulator

## Abstract

Electronic and topological properties of two-dimensional germanene modified by functional group X (X = H, F, OH, CH_3_) at full coverage are studied with first-principles calculation. Without considering the effect of spin-orbit coupling (SOC), all functionalized configurations become semiconductors, removing the Dirac cone at K point in pristine germanene. We also find that their band gaps can be especially well tuned by an external strain. When the SOC is switched on, GeX (X = H, CH_3_) is a normal insulator and strain leads to a phase transition to a topological insulator (TI) phase. However, GeX (X = F, OH) becomes a TI with a large gap of 0.19 eV for X = F and 0.24 eV for X = OH, even without external strains. More interestingly, when all these functionalized monolayers form a bilayer structure, semiconductor-metal states are observed. All these results suggest a possible route of modulating the electronic properties of germanene and promote applications in nanoelectronics.

## 1. Introduction

Due to the novel electronic properties, graphene has attracted plenty of interest since its discovery [1,2,3,4,5], including massless Dirac fermion, high thermal conductivity, and high carrier mobility (200,000 cm^2^/(v s)) [6,7]. Flexibility of graphene is also essential for flexible nanoelectronics, which has produced a lot of products in experiments [8,9,10]. It has a thermal conductivity of 5000 Wm^−1^ K^−1^ at room temperature, offering an advantage for membrane technology [11]. The ambient stability favors sustainable exploitation in nanoelectronics [12]. Moreover, it has half-integer Hall conductance, which indicates promising applications in nanoelectronics. Graphene is also proposed to be a topological insulator (TI) with a band gap opening at the Dirac point by strain or functional group modification with spin-orbit coupling (SOC) [13,14,15]. However, the gap is too small to be detected, let alone having applications at room temperature. In fact, only two-dimensional (2D) TI systems (HgTe/CdTe and InAs/GaSb quantum wells) have been verified in experiments [16,17,18,19]. The prediction and measurement of more 2D TI materials, especially those with large band gaps, is still challenging. These difficulties promote the study of tuning the electronic properties of graphene [20,21]; geometric or chemical modifications are two widely used methods. It also encourages great efforts regarding the search for other honeycomb films composed of heavier group–IV elements such as silicon (Si), germanene (Ge), stanene (Sn) and plumbene (Pb) [22,23,24,25,26,27,28], as well as other group-V films.

Germanene is a promising counterpart of a graphene monolayer, which is a honeycomb monolayer of Ge atoms with a low-buckled (0.84 Å) structure resulted from the weak π-π interaction and distinct coupling of σ and π bonds between Ge atoms [29,30,31,32]. The strength of SOC in germanene is much larger than that in graphene, which leads to a larger band gap at K point, and therefore the expected TI phase may be accessible in experiments. Several remarkable features of germanene have been revealed [28,33,34]. Bianco et al. [35] have synthesized millimeter-scale crystals of hydrogen-terminated germanene from the topochemical deintercalation of CaGe_2_ compound. Zhang et al. [36,37] have investigated the structural and electronic properties of germanene/germanene heterostructures (HTSs). Germanene on MoS_2_ has also been proposed, which is a P-doped semiconductor with a band gap of 24 meV [38].

In the present work, we investigate electronic structures and topological properties of chemically functionalized germanene, GeX (X = H, F, CH_3_, OH), via the use of density functional theory (DFT) calculations. We mainly focus on the effect of both strain and SOC on the band structure. Without considering the effect of SOC, we find that chemical modification will remove the Dirac point at Fermi level and open a direct band gap at Γ point, and which may be well tuned by external strain. Particularly, semiconductor-semimetal phase transition occurs in GeH with increasing strain. Besides this, we also study the bilayer of functionalized germanene, where it can be found that, compared to the band gaps of the isolated germanene monolayer, the band gaps of bilayer are reduced. Functionalization of germanene is a powerful method to produce stable 2D germanene and our results would help to promote the application of germanene in nanoelectronics.

## 2. Methods

All calculations are performed using the density functional theory (DFT) [39] method as implemented in the Vienna Ab initio Simulation Pack (VASP) code [39,40]. The generalized gradient approximation (GGA) with the Perdew–Burke–Ernzerhot (PBE) are used to describe the exchange-correlation energy [41,42], which is developed for the calculations of surface systems. The projector-augmented wave (PAW) method was used to describe electron–ion potential [43]. The DFT-D_2_ methods were used throughout all the calculation to properly take into account the van der Waals (vdW) interaction in the bilayer structures [44]. The energy cutoff of the plane-wave is set to be 500 eV with an energy precision of 10^−6^ eV. A vacuum region of 20 Å was used for the isolated sheet to eliminate any artificial interaction between neighboring super-cells. The Brillouin zone is sampled by using a 9 × 9 × 1 Gamma-centered Monkhorst–Pack (MP) [45] grid. In addition, by using the conjugate gradient method, all atomic positions and the size of the unit cell are optimized until the atomic forces are less than 0.01 eV Å^−1^. SOC is included by a second vibrational procedure on a fully self-consistent basis. A Wannier90 package with the maximally Localized Wannier functions is used to calculate the topological edge state [46]. 

## 3. Results and Discussion

### 3.1. Monolayer Germanene

**Figure 1:**
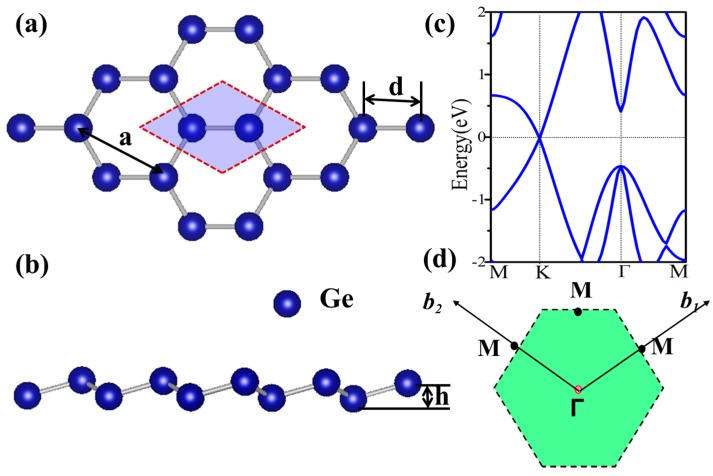
(**a**) Top and (**b**) side view of the atomic stuctures of germanene; (**c**) The energy spectra of germanene; (**d**) The schematic diagram of the first Brillioun zone.

Before studying the functionalized germanene, we first present the geometric and electronic structures of the pristine germanene monolayer, as shown in Figure 1. Germanene has a hexagonal honeycomb structure, and the relaxed lattice parameter is found to be *a* = 4.06 Å and *d* = 2.44 Å. It indicates a weaker π-bonding between Ge atoms, resulting in a low-buckled structure (*h* = 0.69 Å) [47]. The band structure is plotted in Figure 1c, and it is clear that there is a Dirac point at K point near the Fermi level, suggesting a gapless semiconductor. At Γ point, there is a direct gap about 0.86 eV with a two-fold degenerate energy below the Fermi energy, which is mainly attributed to the p_xy_ orbital. The Dirac point is mainly due to the p_z_ orbital, which has a low hybridization with other orbitals. The linear dispersion leads to a high mobility which is important in electronics. To inherit the technology of conventional semiconductor, the opening of a band gap without changing the mobility greatly is expected.

### 3.2. Functionalized Monolayer Germanene

**Figure 2:**
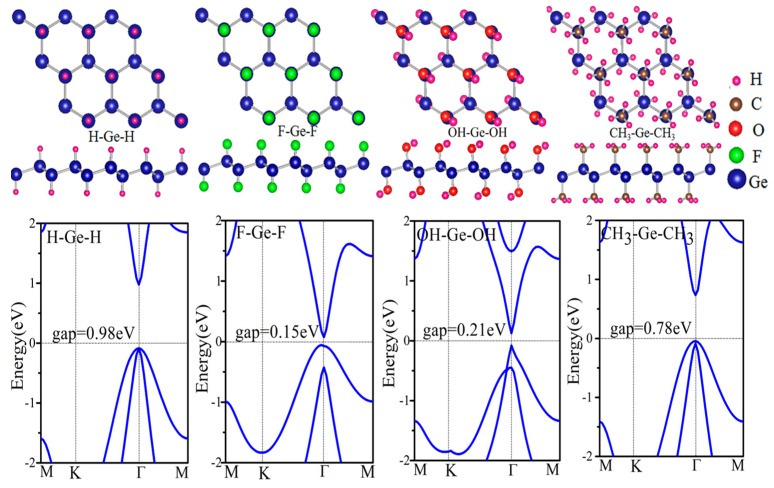
Relaxed geometric structures and the band structures of germanene functionalized with H, F, OH and CH_3_.

**Figure 3:**
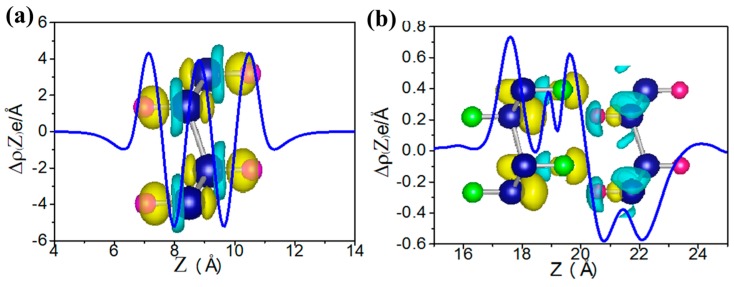
The plane-integrated electron density difference, Δ(Z), for the H–Ge–H (**a**) and the H–Ge–H/F–Ge–F (**b**). Here, for panel (**a**), Δ𝜌(Z) = 𝜌_total_ − 𝜌_germanene_ − 𝜌_H,_ in which 𝜌_total_, 𝜌_germanene_, 𝜌_H_ are the charge densities of H–Ge–H, germanene, and H; while for panel (**b**), Δ𝜌(z) = 𝜌_total_ − 𝜌_H–Ge–H_ − 𝜌_F–Ge–F_ where 𝜌_total_, 𝜌_H–Ge–H_, 𝜌_F–Ge–F_ are charge densities of H–Ge–H/F–Ge–F, H–Ge–H and F–Ge–F, respectively.

Functionalization is a widely used method to tune the electronic properties of 2D film materials. In this work, four four-type functional groups (–H, –F, –OH, –CH_3_) are used. It allows us to design 10 functionalized germanene, four symmetrical (GeX) plotted in Figure 2, and six asymmetrical (X–Ge–X’) ones, as listed in Table 1. Here we denote X–Ge–X’ as the germanene functionalized with radical X on the top side while having radical X’ on the bottom side. We denote GeX as an abbreviation of X–Ge–X’. The structure parameters, formation energies and band gaps are also listed in Table 1. The formation energy is calculated with the formula below.

(1) $$E_{f}=E\left(GeX\right)-E\left(Ge\right)-E(X)$$

where *E*(GeX), *E*(Ge), and *E*(X) are energies of the functional germanene, pristine germanene, and X atoms, respectively, as shown in Table 1. The calculated formation energy *E_f_* is −2.61, −4.25, −3.16 and −4.96 eV/atom for GeH, GeF, GeOH and GeCH_3_, respectively, indicating no phase separation in these systems. In general, we find that the functionalized germanene has larger structural parameters than the pristine one, which is just as expected since electrons will transfer from *Ge* to *X*, which enhances the ion-ion interaction, such as with GeH in Figure 3a.

Figure 2 displays the electronic structure of functional germanene GeX (X = H, F, OH, CH_3_). Note that there is a direct band gap at Γ point with values in the range of 0.15~0.98 eV, while the Dirac point at K point disappears. This is because a strong *σ* bond forms between Ge and X as shown in Figure 3a, where charge distribution is plotted. For pristine germanene, the Dirac point is mainly due to the p_z_ orbital, and there is a direct gap at Γ point, mainly due to the p_xy_ orbital. Their σ bond between Ge and X moves the p_z_ orbital away from the Fermi level, and therefore we have a direct gap at Γ point. For GeH, the direct gap at Γ point is 0.98 eV, a little larger than that of the pristine germanene, while the band structure of GeCH_3_ with a smaller direct gap of 0.78 eV. However, the direct gap is much weakened for GeOH and GeF with gap = 0.15 and 0.21 eV, respectively. The possible reason is that the much-enlarged lattice constants weaken the hopping interaction and therefore result in the band gap between the bonding and anti-bonding state. In this case, the Γ point for the high two valence band is broken in GeF and GeOH; it may lead to the direct gap decrease. In addition, for asymmetrical functionalization germanene monolayer, the band gap is between the corresponding two functionalized structures; such as for OH–Ge–H, the band gap is 0.50 eV, compared with the functionalization structure OH–Ge–OH and H–Ge–H which is 0.21 and 1.06 eV, respectively.

**Figure 4:**
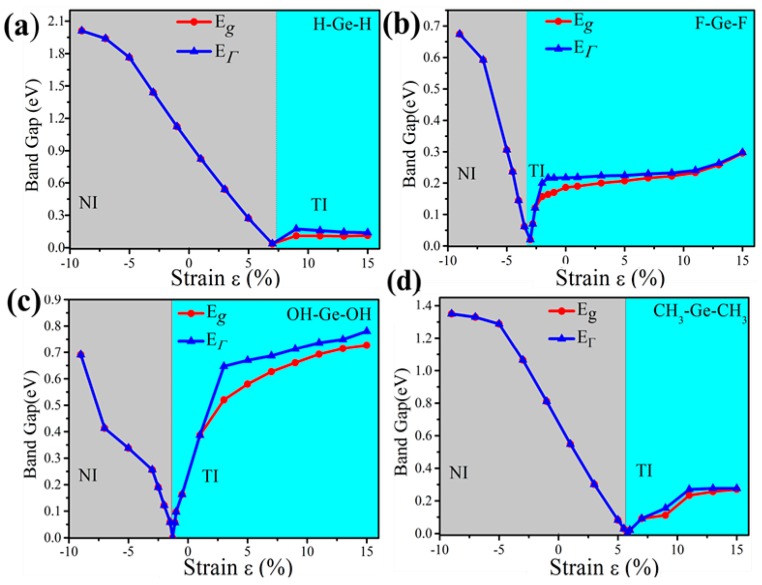
The calculated band gap at Γ point (E_Γ_) and the global band gap (E_g_) of (**a**) GeH; (**b**) GeF; (**c**) GeOH and (**d**) GeCH_3_ with SOC as a function of external strains. Notably, the insets in panel show the trend of band gaps of TI phase as a function of external strain.

**Figure 5:**
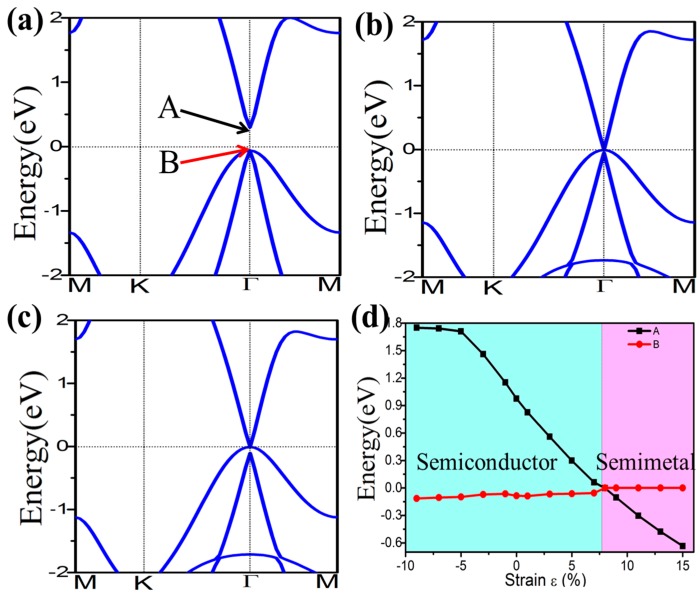
Electronic band structures of GeH under the strain of 0% (**a**); 8% (**b**); and 9% (**c**). Panel (**d**) plots the evolution of the energy level of states A and B under external strain.

External strain is an effective approach to achieve tunable electronic properties. In Figure 4 we plot the evolution of the band gap for the four-symmetrical functionalized germanene. The strain is described by the change of lattice constant *ε* = *(a* − *a*_0_*)/a*_0_, where *a* (*a*_0_) is the lattice constant with (without) strain. In detail, the GeX (*X* = H, F, OH, CH_3_) lattice is expanded uniformly along zigzag and armchair directions in the horizontal plane and relaxed along the *c*-axis. We find that the band gap can be easily tuned, which may be easily realized experimentally. For GeX, the band gap will decrease to zero and reopen with increased strain, as shown in Figure 4. In addition, we take the example of GeH shown in Figure 5. We plot the band structure of GeH under strain 0%, 8% and 9% and find the gap stays closed for strains larger than 8%. Obviously, there is a conduction band and two degenerate valence bands at Γ point near the Fermi level beyond the critical strain, which correspond to the 1D and 2D representations of the symmetry group, respectively. Raising strain will lower the 1D level while lifting the two-fold levels as shown in Figure 5d. In this case, these three bands touch and become three-fold degenerate at Γ point: see Figure 5b. Increasing strain further, thetwo2-fold degenerate levels will lie over the one-fold level: see Figure 5c. Considering the number of electrons, the Fermi level will always cross the touching point, which leads to a semimetal phase.

### 3.3. Topological Properties

In this section, we turn to the effect of SOC on the band structure. We mainly focus on the four-symmetrical functionalized germanene monolayers. Generally, the coexistence of strain and SOC will lead to a topological nontrivial phase in 2D honeycomb lattice, as has been well studied. We investigate the possible topological phase transition by calculating the index Z_2_, edge state and analyzing the inverted band structure.

**Figure 6:**
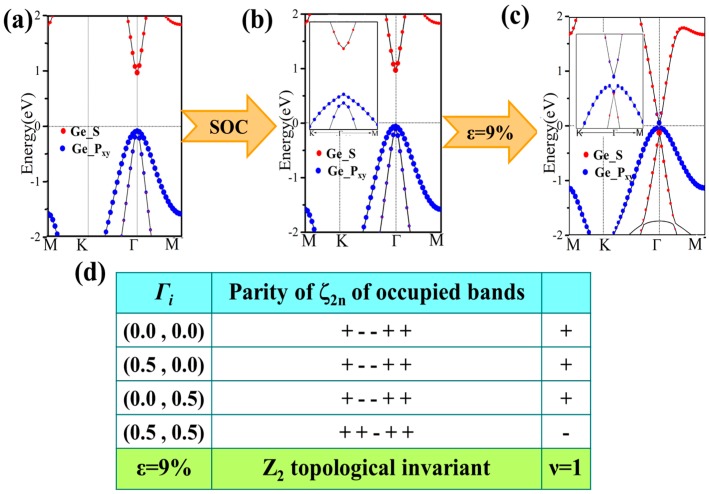
Band structures for GeH without and with spin-orbit coupling SOC (**a**,**b**), and both strain of *ε* = 9% and SOC (**c**). The red and blue dots present the weights of the Ge-s and Ge-p_x,y_ orbitals, respectively. (**d**) Parities of occupied degenerate eigenstates at the time reversal invariant momenta (TRIM) points for GeH. Positive and negative signs denote even and odd parities, respectively.

For GeH, the effects of SOC will only change the band structure slightly: see Figure 6b. It is an insulator with a direct gap of 0.94 eV, a little smaller than that (0.98 eV) without SOC. An obvious difference is that SOC breaks the degeneracy of the first two valence bands at the Γ point. However, there is no topological phase transition in the process, and it still lies in the normal insulator phase. Detailed orbital analysis indicates that two p_xy_ levels and a single s level lie below and above Fermi level at Γ point respectively, which is in the same sequence as in the case with no SOC.

Then, we apply the external strain and reveal its cooperation effect with SOC. We find that the band gap decreases to zero and reopens with increased strain, as is shown in Figure 4a. In addition, the external strain affects the chemical properties of honeycomb nanosystems, which can take a topological phase transition which has been reported by Putz [14]. Thus, we believe that the behavior of band gaps suggests that there may be a topological phase transition caused under external strain. The electronic energy spectra for 9% are shown in Figure 6c. One can see that the orbital sequence at Γ point changes from the original s, p_xy_, p_xy_ to p_xy_, s, p_x__y,_ which leads to an inverted energy band structure and an indirect band gap of 86 meV (a finite band gap of 150 meV opens at Γ) opened under SOC effect. This suggests it is in the TI phase, which is further verified by calculating the topological Z_2_ index, defined as [48].

(2) $$\begin{array}{l} {\left(-1\right)}^{\nu }=\prod_{i=1}^{4}\delta \left(K_{i}\right),\\ \delta \left({Κ}_{i}\right)=\prod_{m=1}^{N}\xi_{2m}^{i} \end{array}$$

*K_i_* is the time reversal invariant momentum in the first Brillouin. For the lattice considered here, there are four time reversal invariant momenta (TRIM) in total, which are Γ (0, 0), M_1_ (0.5, 0), M_2_ (0, 0.5), M_3_ (0.5, 0.5). $\xi_{2m}^{i}$ is the parity of the 2m-th energy level below the Fermi level. In the GeH system, there are 10 valence electrons in a unit cell and therefore N = 5. *δ* is the product of parity eigenvalues at each TRIM. The Z_2_ index *ν* = 0 corresponds to a trivial normal insulator while *ν* = 1 corresponds to a topological insulator. In Figure 6d, we show the parity of the occupied eigenstate at each TRIM in the case with strain *ε* = 9%. The Z_2_ index is calculated to be 1, proving with the prediction of TI phase. 

**Figure 7:**
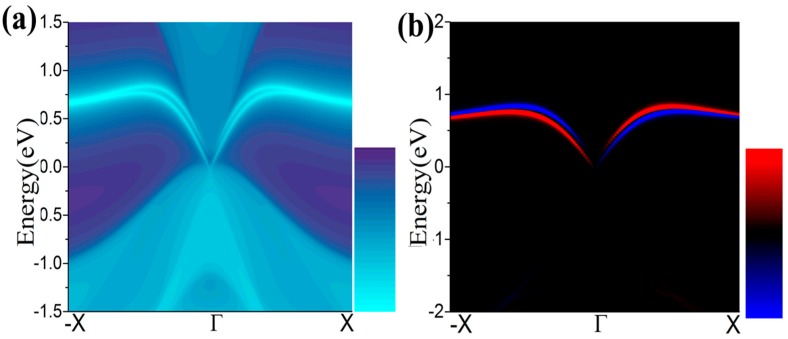
(**a**,**b**) The calculated Dirac edge states, and edge spin polarization, respectively.

One essential characteristic of 2D TIs is the existence of dissipationless edge states protected by time-reversal symmetry (TRS) [49,50]. The density of state (DOS) of a half infinite monolayer and the corresponding spin polarization as illustrated in Figure 7a,b, respectively. There is a pair of gapless edge states in the gap regime connecting the valence and conduction bands. By identifying the spin distribution, as illustrated in Figure 7b, we find that the counter-propagating edge states have opposite spin-polarizations, as expected for the quantum spin Hall (QSH) phase. The transport due to edge states may be used in the design of quantum electronic devices with low dissipation.

**Figure 8:**
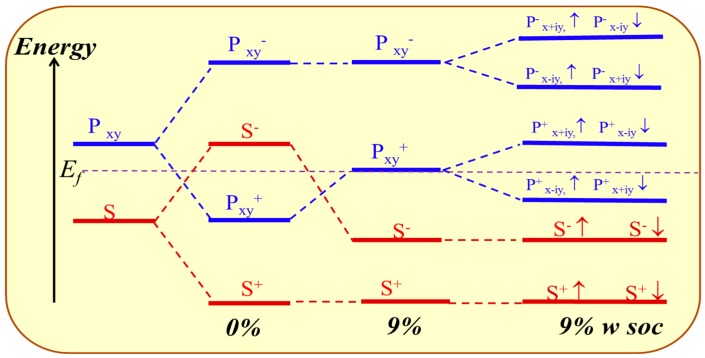
The evolution of atomic s and p_xy_ orbitals of GeH at Γ point described as crystal field splitting and SOC switched on sequence with *ε* = 0% and *ε* = 9%. The horizontal dashed lines indicate the Fermi energy.

An orbital analysis around Fermi level of GeH is necessary to understand the mechanism of transformation from NI to TI. The corresponding orbital analysis is shown in Figure 8, and the relevant bands are dominated by the Ge-4s and Ge-4p_xy_ states. Due to the chemical bonding between adjacent Ge atoms, these states are split into bonding and anti-bonding states, which the superscripts + and − represent the parities of corresponding states, respectively. Here, where the bands near the Fermi level are contributed by the |s^−^> and |p_xy_^+^> orbital, and the |p_xy_^+^> lies below |s^−^> orbital without an inversion of band order. This is because the larger lattice constant of GeH (4.09 Å) results in a weaker s-p hybridization and is according to a smaller energy separation between the bonding and anti-bonding states. When the in-plane strain is applied to *ε* = 9%, the band order of GeH monolayer is changed, the |s^−^> orbital is downshifted while the |p_xy_^+^> orbital is upshifted, leading an s-p band inversion around Fermi level. With the inclusion of SOC, the degeneracy of the level splits into |p, ±3/2> state with a total angular momentum *j* = 1/2, open a band gap. In other words, increasing the lattice constant weakens the interaction between the Ge atoms. This decreases the splitting between the bonding and anti-bonding states, which make signifying that a GeH monolayer with *ε* = 9% is a QSH insulator.

Furthermore, the topological properties of GeX (X = F, OH, CH_3_) monolayers are also studied, which shows similar results as GeH. Figure 4 gives the evolution of band gap at Γ point (E_Γ_) and global band gap (E_g_) against external strain in the presence of SOC. As is shown, it is a direct gap insulator on the left of the critical point, while it becomes an indirect gap for a moderate tensile strain which is especially clear for GeOH. As is well known, it is necessary for the band gap to close and reopen to realize the phase transition from the normal insulator to the topological insulator phase. These demonstrate the emergence of topological nontrivial phase. Also, the calculations of Z_2_ invariants verify a NI (Z_2_ = 0)—TI (Z_2_ = 1) transition.

The critical point for the topological phase transition emerges at a positive tensile strain for GeH (*a* = 4.39Å, *ε* = 8%) and GeCH_3_ (*a* = 4.363 Å, *ε* = 6%) while it lies at a negative compressive strain for GeF (*a* = 4.22 Å, *ε* = −2.8%) and GeOH (*a* = 4.18 Å, *ε* = −1.3%). Thus, the external strain is essential for GeCH_3_ to realize the TI phase while SOC is to open the energy gap, which is identical to the case of GeH. However, TI phase has already formed for GeF and GeOH in the case with no external strain. Particularly, we would point out that the band gap of GeF with no strain is quite large, about 0.19 eV, which is much larger than k_B_T at room temperature (26 meV), indicating edge states in GeF could be measured at room temperature. A tensile strain can only change the band gap of GeF and GeCH_3_ slightly. However, strain will greatly change the band gap of GeOH. It becomes as large as 0.71 eV for GeOH with the strain 9%, such large band gap of TI will be essential for potential applications at room temperature.

### 3.4. Bilayer of Functionalized Germanene

**Figure 9:**
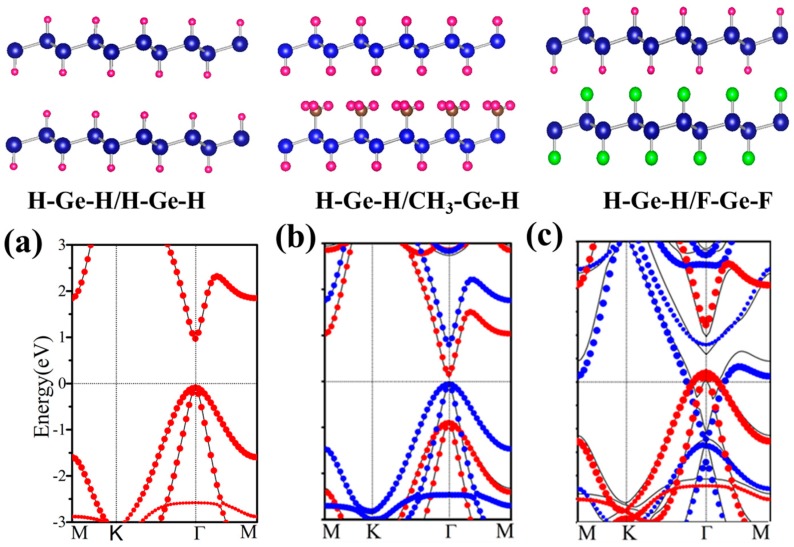
Relaxed geometry structures of H–Ge–H/H–Ge–H, H–Ge–H/CH_3_–Ge–H and H–Ge–H/F–Ge–F and the corresponding energy spectra (**a**–**c**). The black line is the energy dispersion of the bilayer. The red (blue) dots represent the spectra of the isolated up (below) layer. In panel (**a**) red dots coincide with the blue dots.

In this section, we construct AA stacked bilayers of functionalized germanene denoted as X–Ge–X’/Y–Ge–Y’ with monolayer X–Ge–X’ on the top of monolayer Y–Ge–Y’. All structures studied are listed in Table 2 with geometry and electronic parameters. Some of which are plotted in Figure 9. It is expected that it would be possible to further control and improve the electronic properties of functionalized germanene by constructing this bilayer structure.

According to the change of structures, all bilayers can be classified into two classes with one functional group or different functional groups decorated germanene, respectively. If the bilayer is composed of two identical monolayers, band gap and the other geometry parameters change only a little. In Figure 9a, the band structure of H–Ge–H/H–Ge–H is plotted, which is nearly the same as that of H–Ge–H. It indicates that it is a Van der Waals interaction rather than chemical bonding formed between these two layers. For another class, there are different functional groups decorated germanene, such as H–Ge–H/CH_3_–Ge–H, shown in Figure 9b. A direct band gap of 0.18 eV is presented, with its valence band maximum (VBM) and conduction band minimum (CBM) located at Γ point, less than 0.98 and 0.86 eV of H–Ge–H and CH_3_–Ge–H monolayer.

Further analysis indicates that the wave functions of CBM and VBM are located in the H–Ge–H and CH_3_–Ge–H layers, respectively. In Figure 9b, we plot energy spectra of H–Ge–H and CH_3_–Ge–H as red and blue dots, respectively. We find that they fit the energy band of the H–Ge–H/CH_3_–Ge–H bilayer very well. It suggests that no chemical bonding is formed between two layers, and the energy band is nearly a direct combination of the H–Ge–H and CH_3_–Ge–H monolayers.

H–Ge–CH_3_/CH_3_–Ge–H, H–Ge–F/F–Ge–H et al. also have similar results and these results are all in line with those obtained by Li et al. [51] and Pablo et al. [52]. In addition, the second class has another case, such as H–Ge–H/F–Ge–H, H–Ge–H/F–Ge–F, H–Ge–F/CH_3_–Ge–H and H–Ge–HO/CH_3_–Ge-H, where the structure is metal phase. Here, H–Ge–H/F–Ge–F is taken as an example as shown in Figure 9c. It indicates that the Fermi level of one monolayer crosses the energy band of the other monolayer. It means a larger interlayer interaction than in the other structures. To gain further insight into the band gap, we have analyze the plane-integrated electron density differences in Figure 3b. A large charge transfer from H–Ge–H to F–Ge–F monolayer happens, as is expected, consistent with the energy spectra where the original conduction band of F–Ge–F layer lies partly below the Fermi level while the original valence band of the H–Ge–H layer lies partly above the Fermi level. Besides this, we find that the band gap of the bilayer is only slightly changed with the changing of the distance of two component monolayers, which suggests that the interlayer Van der Waals interaction is almost not affected by the change of interlayer distance. Our results show that X–Ge–X’/Y–Ge–Y’ can be conveniently tuned by decorated group, which makes it a promising platform for applications in experiments and electronic devices.

## 4. Conclusions

Based on first-principles calculations, we studied the electronic properties of germanene modified by functional group X (X = H, F, OH, CH_3_). In the monolayer case, we find that the functional group will remove the original Dirac point of pristine germanene at K point and open a gap at Γ point, which can be tuned well by external strain. On the other hand, we have predicted a new class of 2D QSH insulators in GeH monolayers by the external strain. For GeH, external strain can change the band gap significantly and lead to a NI–TI phase transition in the presence of SOC. Further analysis based on orbital analysis indicates that the topology mainly stems from s-p_xy_ orbital of Ge atoms, and the strong SOC is only open a band gap. Lastly, we find that bilayer structures (X–Ge–X’/Y–Ge–Y’) can be conveniently tuned by decorated group. These results suggest a possible route to tune the electronic properties of germanene and provide competitive candidates of TIs for potential applications in nanoelectronics at room temperature.

## Figures and Tables

**Table 1:** Structural parameters (Å), formation energies *E_f_* (eV per unit cell) and band gaps (eV) determined for germanene functionalized with several radicals.

	*a* (Å)	*b* (Å)	Ge–X/Ge–X’ (Å)	Ge–Ge (Å)	*E_f_* (eV)	Gap (eV)
Ge	4.06	4.06	\	2.44	\	0
GeH	4.09	4.09	1.56	2.46	−2.61	0.98
GeCH_3_	4.12	4.12	2.01	2.50	−4.96	0.78
GeOH	4.23	4.23	1.85	2.49	−3.16	0.21
GeF	4.34	4.34	1.79	2.56	−4.25	0.15
H–Ge–CH_3_	4.10	4.10	2.01/1.56	2.49	−3.82	0.86
H–Ge–OH	4.12	4.12	1.84/1.57	2.47	−2.92	0.50
H–Ge–F	4.16	4.16	1.78/1.57	2.45	−3.93	0.49
CH_3_–Ge–OH	4.13	4.15	2.02/1.84	2.51	−4.06	0.44
CH_3_–Ge–F	4.18	4.18	2.02/1.79	2.51	−4.60	0.41
OH–Ge–F	4.33	4.32	1.85/1.79	2.51	−3.68	0.41

**Table 2:** Structural parameters (Å), interlayer interaction energies (eV/Ge atom) and band gaps (eV) determined for bilayer germanene functionalized with several radicals, at full coverage.

Layer1/Layer2	*a* (Å)	*b* (Å)	Interlayer Interaction (eV)	Gap (eV)
H–Ge–H/H–Ge–H	4.09	4.09	0.013	0.96
H–Ge–CH_3_/CH_3_–Ge–H	4.10	4.10	0.030	0.86
CH_3_–Ge–CH_3_/CH_3_–Ge–CH_3_	4.12	4.12	0.011	0.77
H–Ge–F/F–Ge–H	4.17	4.17	0.044	0.45
H–Ge–HO/OH–Ge–H	4.15	4.15	0.109	0.32
H–Ge–H/OH–Ge–H	4.11	4.11	0.002	0.48
H–Ge–H/CH_3_-Ge-H	4.09	4.10	0.013	0.18
H–Ge–HO/F–Ge–H	4.18	4.0	0.042	0.15
H–Ge–H/F–Ge–H	4.13	4.15	0.023	metal
H–Ge–H/F–Ge–F	4.20	4.17	0.034	metal
H–Ge–F/CH_3_–Ge–H	4.15	4.17	0.066	metal
H–Ge–HO/CH_3_–Ge–H	4.11	4.12	0.021	metal
HO–Ge–H/F–Ge–H	4.17	4.17	0.052	metal
